# Biofumigation for the Management of *Fusarium graminearum* in a Wheat-Maize Rotation

**DOI:** 10.3390/pathogens11121427

**Published:** 2022-11-27

**Authors:** Samina Ashiq, Simon Edwards, Andrew Watson, Matthew Back

**Affiliations:** Agriculture and Environment Department, Harper Adams University, Newport TF10 8NB, Shropshire, UK

**Keywords:** biofumigant, sinigrin, head blight, *Brassica juncea*, *Eruca sativa*, *Raphanus sativus*, *Brassica oleracea*

## Abstract

*Fusarium graminearum* is the most important causal agent of head blight in wheat, and stalk and ear rot in maize. A field experiment was conducted to investigate the effect of incorporation of Brassicaceae cover crops on *Fusarium graminearum* in a wheat-maize rotation. Five species belonging to Brassicaceae (*Brassica juncea*, *Eruca sativa*, *Raphanus sativus*, *B. carinata*, *B. oleracea* var. *caulorapa* L.) were used in the field experiment to investigate their potential to suppress *F. graminearum* inoculum in soil, disease incidence in maize and to reduce subsequent mycotoxin contamination in maize. *Brassica juncea* was found to contain the highest glucosinolate concentration in shoots (31 µmol g^−1^). Severity of ear rot and stalk rot in maize was not significantly reduced in the amended plots. Incorporation of *R. sativus* ‘Terranova’ significantly decreased the amount of *F. graminearum* DNA by 58% compared with the cultivated fallow treatment, however the DNA concentration was not significantly different to fallow uncultivated. *Fusarium graminearum* DNA and deoxynivalenol in maize was 50% lower after incorporation of *B. oleracea* var. *caulorapa* L. compared to after fallow treatment but the difference was not significant. The brassica crops used in the present field experiment were not effective in suppressing *F. graminearum*, therefore further studies to optimise the current approach are recommended.

## 1. Introduction

Wheat is one of the most important agricultural crops globally. In terms of dietary intake, it is the most important staple commodity [1] and the most widely grown crop with more than 219 million ha grown worldwide in 2020 [2]. Maize is the third leading staple after rice and the most produced crop in the world [1]. An average global production of over 1 billion t of maize has been reported for the eight-year period (2013–2020) [2]. In 2020, the area under maize cultivation in the EU was 9 million ha producing 67 million t [2]. A major disease problem in wheat and maize is caused by the fungal pathogen *Fusarium graminearum* which causes Fusarium head blight in wheat and ear and stalk rot in maize [3,4]. *Fusarium graminearum* infection causes shrivelled kernels, poor grain quality and mycotoxin contamination. The harvested grains which may appear normal and healthy can still be contaminated with mycotoxins [5]. The mycotoxins of most concern produced by *F. graminearum* are deoxynivalenol (DON) and zearalenone (ZON). Deoxynivalenol induces vomiting, anorexia and reduced food intake in humans and animals. It also has hepatotoxic, immunotoxic and neurovirulent effects [6]. In contrast, ZON mainly has estrogenic effects in humans and animals, causing reproductive and fertility disorders and premature puberty [7]. Additionally, ZON may also cause hematotoxicity, genotoxicity, hepatotoxicity and immunotoxicity [8]. In addition to health consequences, these mycotoxins are responsible for significant economic losses. In the US, Fusarium head blight resulted in yield losses worth $1.176 billion in 2015/16 [9]. For consumer protection, the European Commission has set legislative limits for ZON at 100 µg kg^−1^ and for DON at 1250 µg kg^−1^ in wheat. In maize the limits are set at 350 µg kg^−1^ and 1750 µg kg^−1^ for ZON and DON, respectively [10]. Contamination exceeding legal limits of DON and ZON were reported in 13% and 29% of wheat samples, respectively, in England in 2008 [11]. In Croatia, 50% and 28% samples of maize collected in 2010 contained DON and ZON, respectively, at concentrations above the legal limits [12]. Results from a recent survey conducted in Luxembourg [13] showed that concentrations of DON above the legal limits were detected in 5% wheat samples collected during a 12-year period (2007–2018). In the same study, 15% of wheat samples from 2018 contained DON levels above legal limits. 

There are different factors that are important for the establishment and development of Fusarium head blight. These include humid weather conditions during anthesis [14] and density of residues from previous crops, affecting the severity of the disease and DON contamination [14]. Maize as a preceding crop is known to be an important risk factor for Fusarium head blight in the subsequent wheat [15] as large quantities of infected residues that serve as substrate for *F. graminearum* remain after harvest [16]. *Fusarium graminearum* survives as mycelium on crop residues and produces ascospores (sexual) and conidia (asexual). These spores and hyphal fragments, air-dispersed and water-splashed, serve as source of inoculum in the subsequent cereal crop [17,18].

Currently, *F. graminearum* is managed through triazole fungicides such as prothioconazole and tebuconazole, however due to the endocrine-disrupting properties of these fungicides, serious concerns have been raised [19]. Due to these safety concerns, their limited effectiveness and high selection pressure for fungicide resistance [20], there is a need to explore an alternative control strategy. Biofumigation as a crop protection strategy has recently gained interest. This practice involves growing short term brassica crops, followed by maceration of the plant tissue and rapid incorporation into the soil. Inhibitory volatile substances, particularly isothiocyanates (ITC) are produced as a result of damage to brassica plant tissue causing suppression of soil-borne pests and diseases [21]. The suppressive effect of biofumigation is generally attributed to the toxicity of isothiocyanate (ITC) which is produced as a result of glucosinolate (GSL) hydrolysis by myrosinase enzyme upon plant tissue disruption. However, the application of green manure also increases the organic matter content in the soil which supports soil saprophytes, thus enhancing their competition and antagonism effects. Additionally, toxic compounds released during the decomposition of the organic matter may contribute to the suppressive activity [22,23,24].

Whilst biofumigation has attracted significant interest, research on its potential application for reducing the inoculum of *Fusarium* species affecting cereals is scarce. Previously, fungal pathogens of potato were suppressed using biofumigation in the glasshouse and in the field [25]. Incorporation of *Brassica juncea* was shown to reduce disease incidence and severity of powdery scab (*Spongospora subterranea*) and common scab (*Streptomyces scabies*) by 40% and less than 20%, respectively. Recently, Drakopoulos et al. [26] applied the mulch of *Sinapis alba* and *B. juncea*, in a cut-and-carry approach, to *F. graminearum*-infected wheat plots; Fusarium head blight incidence was significantly reduced by 58% in the first year by *S. alba* and 18% by *B. juncea* in the second year, in field experiments performed over two years.

Following encouraging results from studies conducted under laboratory conditions [27,28], a field experiment was undertaken to investigate the potential of incorporation of brassica crops on suppressing *F*. *graminearum* inoculum in a wheat-maize rotation.

## 2. Materials and Methods

### 2.1. Experimental Set Up

From June 2018 to December 2019, a field experiment was conducted at ‘Heaford’ field, Harper Adams University, Newport, Shropshire, UK (52°47′04.0″ N 2°26′04.9″ W) in a 72 × 18 m area. The soil at this site is a sandy clay loam (62% sand, 18% silt, 20% clay) with organic matter content 6%, pH 5.9 and available sulphate 20.75 mg L^−1^. The field experiment was designed for Brassicaceae cover crops incorporation in a wheat-maize rotation. The experiment comprised six replicates of eight treatments in a randomised complete block design and each plot was 27 m^2^ (9 × 3 m). Treatments included *Brassica juncea* ‘Brons’ (Indian mustard), *Eruca sativa* ‘Trio’ (rocket), *Raphanus sativus* ‘Bokito’ (oilseed radish), *R. sativus* ‘Terranova’, *B. carinata* ‘Cappucchino’ (Ethiopian mustard), *B. oleracea* var. *caulorapa* L. ‘Kolibri’ (kohlrabi), fallow cultivated and fallow uncultivated. ‘Fallow cultivated’ were plots in which no crops were grown, but flail, rotovator and roller were used on these plots similar to brassica plots. Alternatively, ‘fallow uncultivated’ had no crops and no machinery used except roller. Seeds of all Brassicaceae were supplied by RAGT seeds, UK except *B. oleracea* var. *caulorapa* L. ‘Kolibri’ supplied by Elsoms, UK. All crops were established and maintained according to standard crop husbandry practices at Harper Adams University, Shropshire, UK.

### 2.2. Semi-Artificial Infection of Wheat

**Figure 1:**
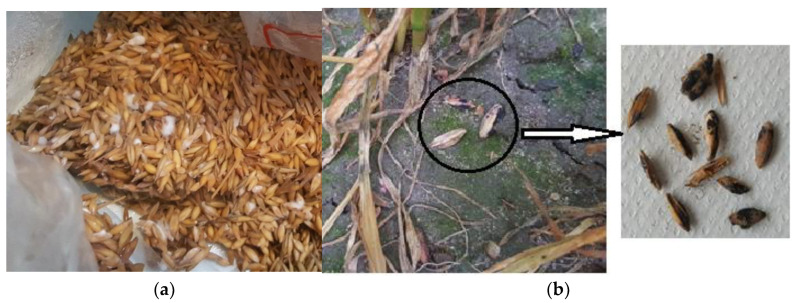
Experimental set up of the study: (**a**) Oats inoculated with *Fusarium graminearum* conidial suspensions after incubation, (**b**) Perithecia of *F. graminearum* formed on the oats on the soil surface five weeks after inoculation of wheat plots.

One kg of oat was soaked in 200 mL distilled water for 2 h, drained and autoclaved in a 78 × 40 cm autoclavable bag [VWR (129-0581)] at 124 °C for 1 h. After 24 h the oats were re-autoclaved at 124 °C for 1 h and allowed to cool. Oat broth was prepared by adding 1 g oat flour to 100 mL sterile distilled water in 250 mL flasks. A cotton plug was inserted into the neck of the flask before it was covered with tin foil and autoclaved at 121 °C for 15 min. Five 7 mm mycelial plugs, taken from a five-day old colony of *F. graminearum* growing on potato dextrose agar (PDA) (Merck, KGaA, Darmstadt, Germany), were added to the flask containing oat broth and incubated on an orbital shaker at 120 rpm for 7 days. A 100 mL of the oat broth was added to 1.25 L potato dextrose broth (PDB) (Oxoid, Basingstoke, UK) and mixed. A total amount of 100 mL of the PDB mixture containing *F. graminearum* was added to each autoclaved oat bag, mixed well and incubated for 3 weeks before field inoculation (Figure 1a). Bags were agitated every few days to avoid clumping of the inoculum. All incubations were at room temperature (ca. 18 °C). A total of 40 kg of *Fusarium* inoculum was prepared with an equal amount for each of five fungal strains (FG2556, FG2560, FG2481, FG2498, FG2502). All strains were isolated from Fusarium head blight-infected wheat samples collected in 2016 and were supplied by Dr Phil Jennings, Fera Sciences Ltd (York, UK). All strains were identified with species-specific PCR [29]. Bags were mixed to create a bulk bag of inoculum for each experimental block with an equal mix of each strain.

Winter wheat ‘Shabras’ was sown in early October 2017 and maintained using standard agronomy. Wheat at early stem-extension stage (Zadoks GS31 [30]) was inoculated manually by dispersing the oat inoculum at 25 g m^−2^. Perithecia of *F. graminearum* were observed on the oat grain inoculum five weeks after application (Figure 1b).

The disease incidence in the field was measured by counting the number of ears with typical Fusarium head blight symptoms of partially bleached ears, at medium milk (GS75). Wheat ears in a 60 × 120 cm quadrat were observed at 24 different locations in a central area (60 × 17 m) of the trial area at 5–12 m apart. Disease incidence was expressed as the percentage of symptomatic wheat ears. 

Post-harvest, wheat debris were collected to test presence of *F. graminearum*. Chaff, rachis and straw separately were randomly collected from the field, additionally the three type of wheat debris were also collected from each block. Straw and rachis were cut into 1 cm pieces. One hundred pieces of each type of wheat debris (collected from whole field) and 100 pieces of wheat debris from each block (collected block-wise) were plated out on modified Czapek Dox iprodione agar media [31] plates with five pieces per plate. Plates were incubated at room temperature (ca. 18 °C) for 12–15 days. *Fusarium graminearum* incidence in the inoculum pieces was recorded as presence of *F. graminearum* colony growth based on the characteristic reddish pigmentation. For confirmation, some of the assumed *F. graminearum* colonies were sub-cultured on PDA and incubated at room temperature (ca. 18 °C) for 14 days. The conidia were harvested as described in Ashiq et al. [27] and confirmed as *F. graminearum* based on spore morphology [32].

### 2.3. Biofumigants-Biomass, Glucosinolate Analysis, Incorporation

Following wheat harvest in late July 2018, cover crops were sown on 13 August 2018 using the recommended seed rate (Table 1) with 100 kg ha^−1^ nitrogen and 25 kg ha^−1^ sulphur applied to the seedbed. On 19 November 2018, the brassica crops, at 25% flowering, were flailed and rotovated. Following incorporation, the soil surface was rolled to reduce soil porosity. Flail and rotovator were used on all plots except uncultivated fallow plots.

**Glucosinolate analysis**: Prior to incorporation, plants for GSL analysis were sampled and processed as described by Ngala et al. [33]. Briefly, three samples from each plot were randomly selected, uprooted and processed carefully, roots and shoots separately flash frozen in liquid nitrogen, stored at −80 °C until freeze dried (GVD6/13 MKI freeze dryer; GIROVAC Ltd., North Walsham, UK). Freeze-dried samples were milled and stored at −18 °C. Samples collected from four blocks (2, 3, 4, 6) were sent to NIAB Labtest, Cambridge, UK where GSL analysis was performed following ISO 9167 “Rapeseed and rapeseed meals—Determination of glucosinolates content—Method using HPLC” [34].

**Biomass**: Prior to incorporation, plants from two 0.33 m^2^ quadrats (within central 1 × 5 m area of plot) were uprooted, taken to the laboratory, separated into roots and shoots and weighed fresh. The plant samples were then placed in a forced air oven, for drying at 100 °C for 48 h, to calculate the dry weight.

### 2.4. Assessment of Fusarium graminearum Inoculum Buried in Sachets Post-Incorporation of Brassica Biofumigants

Blind oat spikes from oat screenings (Morning Foods, Crewe, UK) were used as artificial crop debris. One kg of oat screenings was soaked in distilled water for 12 h before autoclaving in a 78 × 40 cm autoclavable bag [VWR (129-0581)]. The blind oat spikes were re-autoclaved after 24 h and allowed to cool. Spore suspension was prepared as described in Ashiq et al. [28]. A 100 mL of the spore suspension was added to the bag, mixed well and incubated for 10–12 days in dark. The inoculum was confirmed to be infected with *F. graminearum* by plating on PDA prior to using in the experiment. Square 12.5 cm^2^ fine mesh (voile) sachets were prepared using a heat sealer. A 50 cm nylon string with label tag was tied to each sachet. Six grams of the above prepared inoculum, which contained more than 100 pieces of inoculum, were added to each of the 288 sachets (six sachets per plot) and sealed with heat-sealer. Immediately after brassica incorporation, six sachets of the inoculum were buried at 10 cm depth in a central area (1 × 4 m) of each plot, at intervals of 1–2 m apart. Pegs were tied to the nylon string of the sachets to enable easy location and removal of the sachets later. From each plot, three sachets were removed 8 and 16 weeks after their burial and transported to laboratory to assess for *F. graminearum* incidence. One hundred inoculum pieces from each sachet were plated out) (10 pieces per plate) on modified Czapek Dox iprodione agar media [31] and incubated at ca. 18 °C. After 12–15 days, *F. graminearum* growth was recorded based on the characteristic reddish pigmentation of *F. graminearum* colonies and the *Fusarium* incidence was calculated.

### 2.5. Maize Assessment

#### 2.5.1. Disease Assessment and *Fusarium graminearum* Incidence

Maize ‘Ambition’ was sown mid-April 2019. In December 2019, when the maize had a grain moisture content of 44%, 30 plants were randomly sampled from a 1 × 6 m area in the centre of each plot. Primary cobs were handpicked and stalks were cut in lateral direction above roots and above the second node from bottom. Samples were transported to laboratory, where ears were husked and the ear surface covered with mycelium was visually assessed for ear rot. Stalks were dissected and the infected area of lower node, upper node and internode was visually assessed for stalk rot (red staining). Rot that was not caused by *Fusarium* was not assessed. Symptoms of *Fusarium* infection were assessed according to a seven-class disease severity rating scale of 0 (no infection) to 6 (infection symptoms close to 100% of plant surface) [35] and disease severity % was calculated by McKinney Index [36].

One row of kernels from each of the 30 ears were removed by hand, mixed, before 100 representative kernels were used to assess *Fusarium* incidence. The remaining kernels were placed in perforated plastic bags and dried at 60 °C with forced ventilation for mycotoxin and molecular analysis. Maize kernels (100 per plot) were surface disinfected with sodium hypochlorite (1.2% available chlorine) containing 0.05% Tween 20 for 3 min, rinsed with sterile distilled water thrice and plated out on PDA plates supplemented with streptomycin sulphate (130 mg L^−1^). Plates were incubated at room temperature (ca. 18 °C) for 10–12 days and *F. graminearum* mycelial growth was recorded based on the characteristic reddish coloured colonies. *Fusarium graminearum* growth was confirmed as described above. *Fusarium* incidence was calculated as mean of the number of kernels infected.

#### 2.5.2. Mycotoxin and Molecular Analysis

The dried maize kernels were ground with a mill (Retsch ZM200, Germany; 1 mm mesh size) and samples were stored at −18 °C until further processing. 

DON was quantified in 8 g of milled flour using a DON ELISA assay (Agraquant, Romer Lab Diagnostic, Austria) according to the manufacturer’s instructions. Additionally, the flour was analysed for *F. graminearum* DNA. DNA was extracted from flour using a cetyltrimethylammonium bromide (CTAB) buffer and quantified by spectrophotometry as detailed in Edwards et al. [37] and diluted to 20 ng µL^−1^. DNA was first amplified with ITS 4 and 5 primers [38] with an annealing temperature of 50 °C [39]. At this temperature, both fungal and plant DNA are amplified, hence it was confirmed that amplifiable DNA is present within each sample. PCR products were observed on a 2% agarose gel in Tris-acetate-EDTA (TAE) buffer using GelRed loading buffer (Biotium). *Fusarium graminearum* DNA was then quantified using *Tri5* primers as described by Edwards et al. [37] by SYBR green qPCR method. EvaGreen master mix (Biotium) was used and 10-fold dilutions of 10 ng µL^−1^ of *F. graminearum* DNA were used as standards. The program used had 40 temperature cycles of 95 °C for 15 s, 62 °C for 15 s, 72 °C for 30 s and 82 °C for 10 s with fluorescence measured at 82 °C. The first cycle had an extra 12 min at 95 °C. *Fusarium graminearum* DNA was measured relative to the total (plant and fungal) DNA extracted (pg *F. graminearum* DNA ng^−1^ total DNA).

### 2.6. Statistical Analysis

Data were subjected to one-way analysis of variance using Genstat^®^ (20th edition) statistical software. Data from assessment of *F. graminearum* in inoculum (sachets) and maize kernels were logit transformed and angular transformed, respectively. Data from DON and *F. graminearum* DNA in maize were log10 transformed. Significant differences between treatments were determined using post hoc Tukey’s test (*p* = 0.05).

## 3. Results

### 3.1. Fusarium graminearum in Wheat

Fusarium head blight incidence was 2.6% in the winter wheat crop, assessed on the medium milk (GS75). Post-harvest, *F. graminearum* infection in chaff, rachis and straw were found to be 92%, 89% and 59%, respectively. *Fusarium graminearum* infection was detected in wheat debris from all blocks, ranging from 90 to 98%.

### 3.2. Biomass, Glucosinolate Concentrations of Brassicas

**Figure 2:**
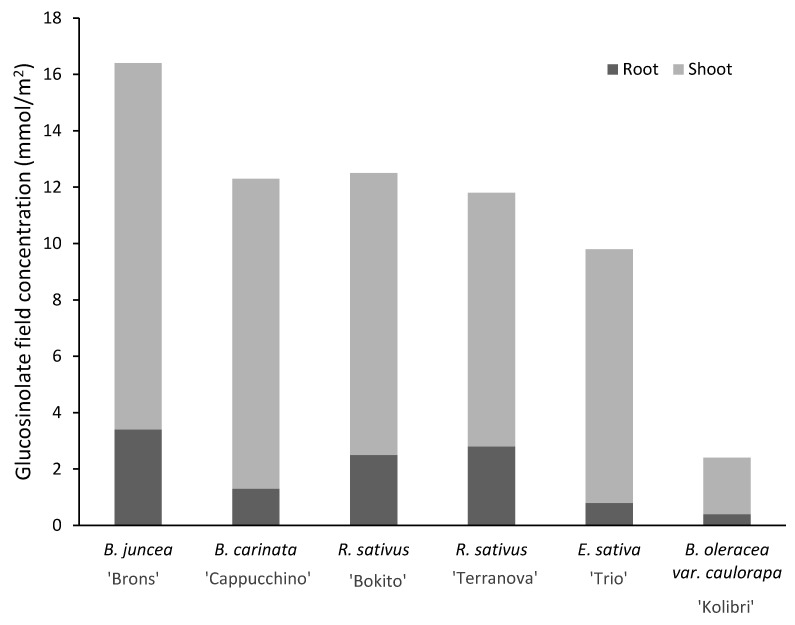
Expected glucosinolate concentration per m^2^ estimated from the biomass and glucosinolate concentrations detected in brassica plants at 13 weeks after planting.

The total above-ground biomass (fresh weight) of the brassicas ranged from 19 t ha^−1^ (*B. oleracea* var. *caulorapa* L. ‘Kolibri’) to 79 t ha^−1^ (*R. sativus* ‘Bokito’) (Table 2). The two cultivars of *R. sativus* produced similar biomass (85 and 89 t ha^−1^ fresh weight; 6.5 and 7.4 t ha^−1^ dry weight). *Brassica juncea* ‘Brons’ produced 48 t ha^−1^ of above-ground biomass (fresh weight). The GSL profile found in the brassicas varied both qualitatively and quantitatively (Table 3). *Brassica juncea* was found to contain the highest total GSL concentration among all the brassicas tested. The predominant GSL of *B. juncea* was sinigrin, precursor of allyl isothiocyanate (AITC), and it comprised 85% of the total GSL content of the shoot tissue. On the other hand, sinigrin made up 79% of total GSL content of shoot tissue of *B. carinata*, occurring at a concentration of 13.1 µmol g^−1^. The predominant GSL found in root tissue of *R. sativus* was glucoraphasatin (4-methylsulfanyl-3- butenyl ITC- or raphasatin-precursor). The GSL concentration expected per area of field was estimated (Figure 2). For *B. juncea*, the total GSL concentration (shoots) expected in the field was estimated to be 13 mmol m^−2^ and that for *B. carinata* was 11 mmol m^−2^.

### 3.3. Effect of Brassica Incorporation on F. graminearum Inoculum in Sachets 

For both sets of sachets (removal after 8 and 16 weeks after brassica incorporation), the percentage incidence of *F. graminearum* ranged from 94 to 98% for all treatments (Table 4). The effect of brassica incorporation on *F. graminearum* inoculum was not significant (*p* = 0.677 and *p* = 0.074 after 8 and 16 weeks, respectively). CV% and SED for set 1 (removal after 8 weeks) were 31.8 and 0.57, respectively, and that for set 2 (removal after 16 weeks) were 20.4 and 0.37.

### 3.4. Effect of Brassica Biofumigation on Fusarium graminearum Diseases in a Subsequent Maize Crop

#### 3.4.1. Disease Severity and *Fusarium graminearum* Incidence in Maize

**Figure 3:**
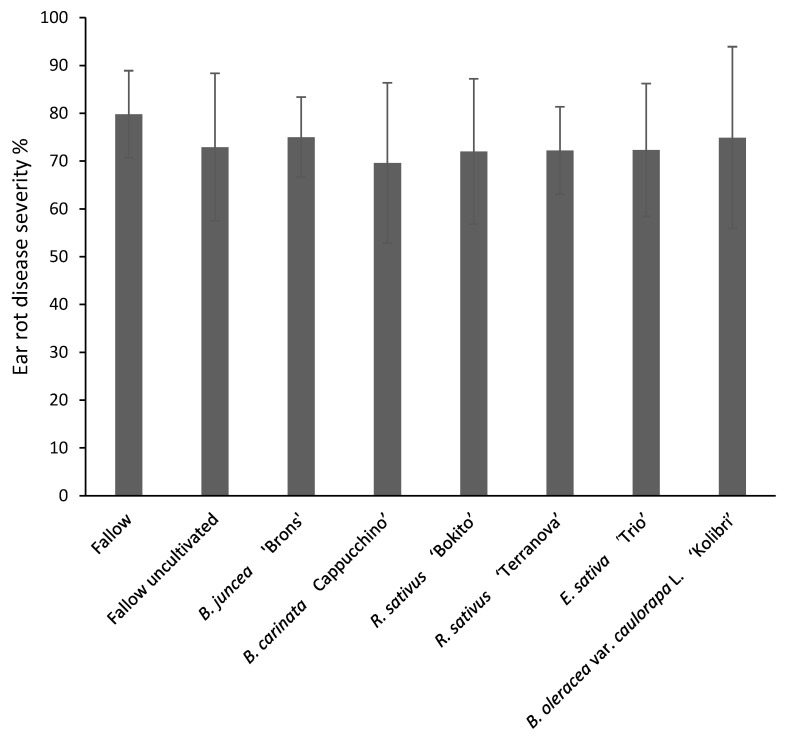
Ear rot severity in maize grown in brassica-incorporated plots preceded by *Fusarium graminearum*-inoculated wheat. Ears were visually assessed according to a 7-class disease severity rating scale of 0 (no infection) to 6 (infection symptoms close to 100% of plant surface) and disease severity % was calculated by McKinney Index. Analysis of variance showed no significant difference between treatments (*p* = 0.579, CV% = 11.2, SED = 4.75). Error bars represent standard deviations.

**Figure 4:**
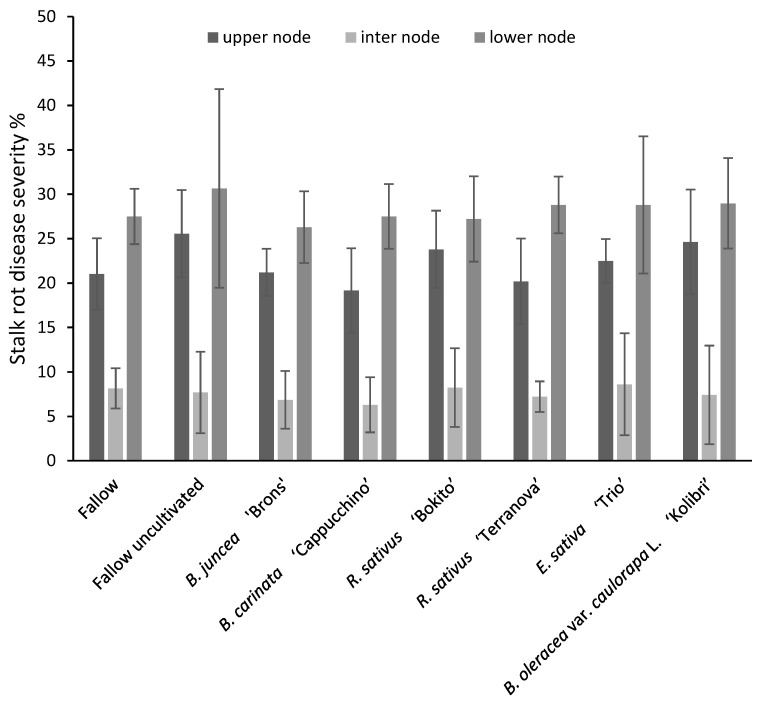
Stalk rot severity in maize grown in brassica-incorporated plots preceded by *Fusarium graminearum*-inoculated wheat. Upper node, lower node and internode of stalks were visually assessed according to a 7-class disease severity rating scale of 0 (no infection) to 6 (infection symptoms close to 100% of plant surface) and disease severity % was calculated by McKinney Index. Analysis of variance showed no significant difference between treatments (upper node: *p* = 0.093, CV% = 17.8, SED = 2.287; inter node: *p* = 0.971, CV% = 50.6, SED = 2.21; lower node: *p* = 0.942, CV% = 21, SED = 3.418). Error bars represent standard deviations.

**Figure 5:**
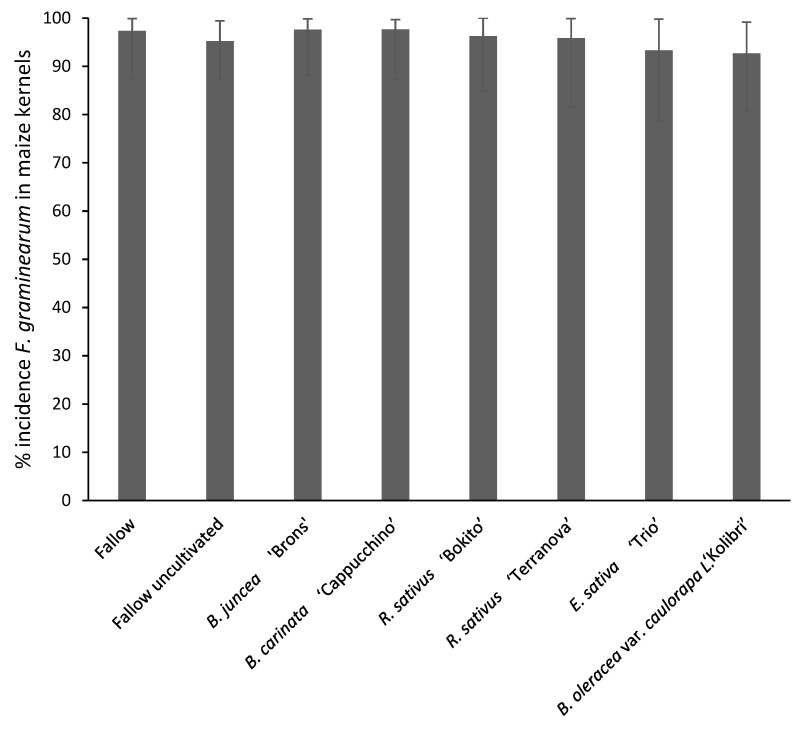
Percentage incidence of *Fusarium graminearum* in kernels of maize grown in brassica-incorporated plots preceded by *Fusarium graminearum*-inoculated wheat. Representative kernels were plated out on media and assessed for *F. graminearum* growth. Analysis of variance showed no significant difference between treatments (*p* = 0.411, CV% = 8.1, SED = 3.642). Error bars represent standard deviations.

Disease severity of ear rot ranged from 70% in *B. carinata*-treated plots to 80% in fallow plots (Figure 3). However, analysis of variance showed no significant effect of brassica incorporation on ear rot severity (*p* = 0.579). Disease severity of stalk rot in the upper, lower and internodes of maize stalks is shown in Figure 4. The lowest disease severity percentage in upper nodes was found in *B. carinata* treatment (19%) and the highest (26%) in fallow uncultivated. The disease severity percentage in internodes was similar (6–9%) in all treatments. The effect of brassicas on stalk rot severity was insignificant (*p* = 0.942). Maize kernels tested for *F. graminearum* incidence were found to be infected in all treatments ranging from 93 to 98% with no significant effect (*p* = 0.411) between the treatments (Figure 5).

#### 3.4.2. Assessment of Deoxynivalenol and *Fusarium graminearum* DNA in Maize

**Figure 6:**
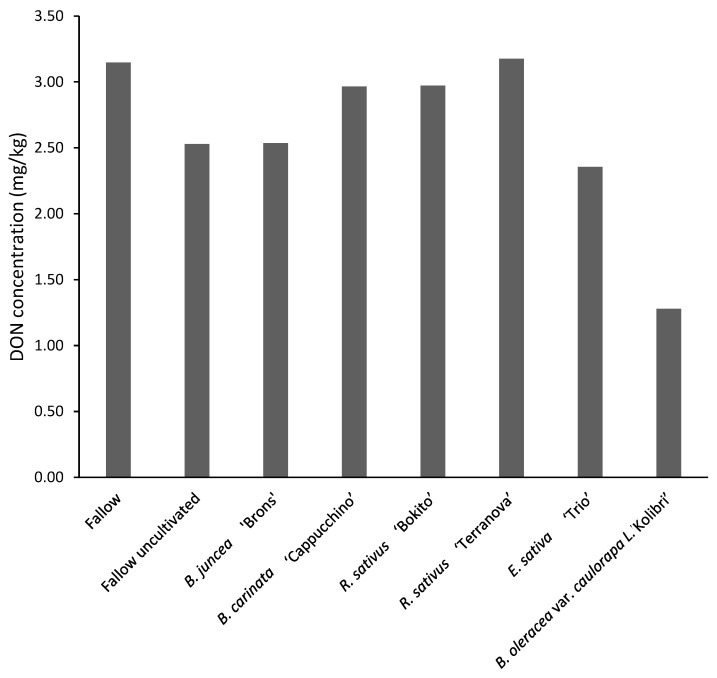
Deoxynivalenol (DON, mg kg^−1^) concentration in maize grown in brassica-incorporated plots preceded by *Fusarium graminearum*-inoculated wheat. Analysis of variance showed no significant differences between treatments (*p* = 0.635, CV% = 90.8, SED = 0.211).

**Figure 7:**
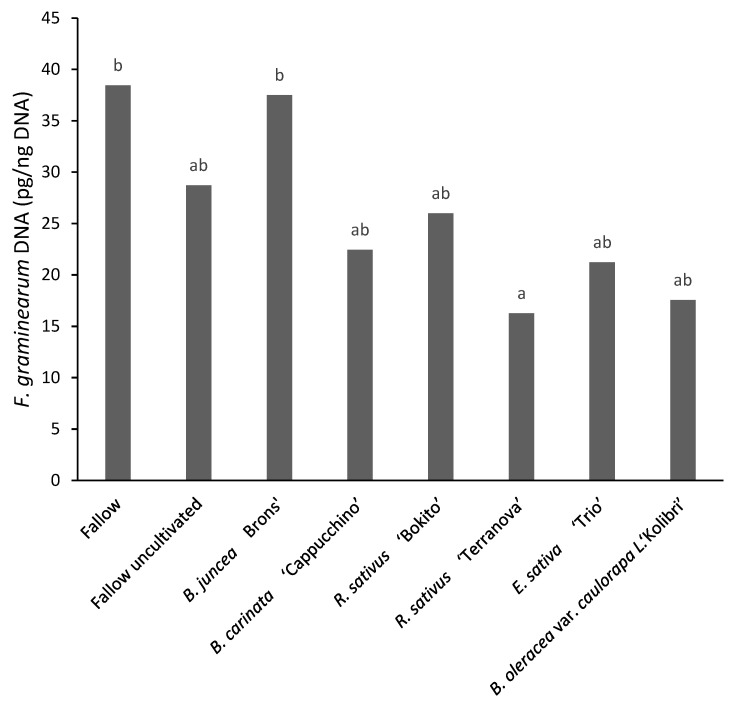
*Fusarium graminearum* DNA content in maize grown in brassica-incorporated plots preceded by *Fusarium graminearum*-inoculated wheat. Different letters indicate significant differences according to post hoc Tukey’s test at 5% significance level (CV% = 13.8, SED = 0.111).

All samples were found to be contaminated with DON, with 67% samples exceeding the legal limit of 1.75 mg kg^−1^ DON in maize for human consumption (Figure 6). The effect of brassicas on DON contamination was found to be non-significant (*p* = 0.635). The concentrations in the 48 samples ranged from 0.51 to 14.33 mg kg^−1^. Deoxynivalenol concentrations in maize was 50–60% lower after incorporation of *B. oleracea* var. *caulorapa* L. compared to after the two fallow treatments. *Brassica juncea*-treated plots were found to have the highest variation of DON concentrations in the maize flour with values ranging from 0.55 to 14.33 mg kg^−1^ (Figure 7). Incorporation of *R. sativus* ‘Terranova’ significantly decreased (*p* < 0.05) the amount of *F. graminearum* DNA by 58% compared with the fallow cultivated treatment, however the level was not significantly different to fallow uncultivated. Maize grown after *B. oleracea* var. *caulorapa* L. treatment also had *F. graminearum* DNA 55% lower compared to maize grown after the cultivated fallow treatment, but the difference was not significant.

## 4. Discussion

In the present two-year study, biofumigation was not effective in suppressing *F. graminearum* in the soil and in the following maize crop. Following incorporation of brassicas, *F. graminearum* infection in maize was not significantly lower compared to control (fallow) plots. This is in contrast to results from previous work in the laboratory [27,28] indicating brassicas could have a suppressive effect on *F. graminearum* under field conditions.

Biofumigant brassicas have been effective in suppressing soil-borne pathogens in studies conducted elsewhere. For example, Subbarao et al. [40] reported suppression of Verticillium wilt in cauliflower by broccoli residue incorporation in comparison to metam sodium, chloropicrin and other control treatments. The soil population of *Verticillium dahliae* microsclerotia was reduced by 50–75% following incorporation of broccoli, compared to pre-treatment levels. Drakopoulos et al. [26] reported 40–50% DON reduction in wheat flour by using *B. juncea* and *S. alba* mulch in field experiments. While these studies have demonstrated the effectiveness of biofumigation in suppressing disease, lack of disease control has also been reported. In a field experiment [41], where *F. oxysporum*, *Rhizoctonia solani* and *V. dahliae* inoculum were buried in soil previously amended with broccoli at 34–38 t ha^−1^ (fresh weight), no suppression was recorded. Similarly, in another study [42], incorporation of *B. juncea* and *B. napus* in field was not effective in suppressing *F. oxysporum* or *Pythium* spp. populations in soil.

There could be a number of factors that might have impacted the efficacy of biofumigation in the present study. Soil temperature has a significant impact on ITC production [43]. Previously, soil amended with cabbage residues was analysed for volatile production [44]. It was reported that the concentration of volatiles in the headspace were higher in heated, amended soils than in non-heated amended soils. In the present study, when the plants were incorporated in November, mean soil temperatures at ~10 cm deep were 7.7 °C which might not have been high enough to favour effective ITC production. Moreover, due to dry weather conditions, ITC production might not have been sufficient. The average rainfall for November 2018 was 36 mm which was half the amount compared to the average rainfall for November (72 mm) for the previous five-year period (2013–2017) according to the data from nearest weather station at Shawbury, UK [45]. Presence of sufficient moisture is important to enable myrosinase activity for production of GSL hydrolysis products [46]. In a field study conducted by Matthiessen et al. [47], addition of 42 mm of water to *B. juncea* plant material resulted in a 7- to 10-fold increase in ITC concentrations in soil compared to where no water was added. Wang and Mazzola [48] amended soil with *B. juncea* and *S. alba* seed meal in jars and evaluated AITC emission in the headspace. They reported that AITC production elevated (~0.05–0.265 µg g^−1^ soil) with an increase in soil temperature from 10 °C to 30 °C and increase in moisture level from −1000 kPa to −40 kPa. Another factor that might have affected the activity of ITC could be the organic matter content in the soil. Gimsing et al. [49] demonstrated that organic matter content is the main sorbent of ITC. Organic matter in arable soils is usually at 2–4% [50] compared to which the organic matter content in soil at the field experiment site was higher at 6%. Sorption of ITC to the organic matter in soil might have resulted in reduced activity of ITC.

In a study investigating the ITC-release potential of brassicas under field conditions, *R. sativus*, at a seed rate of 20 kg ha^−1^ produced a biomass of 71–74 t ha^−1^ and an estimated 31–45 mmol m^−2^ GSL [51]. Conversely in the present study, at a similar seed rate, *R. sativus* produced similar biomass (74–79 t ha^−1^) but the expected GSL concentration in the field was much lower (9–10 mmol m^−2^). The difference in GSL concentrations could be due to difference in soil conditions such as temperature and moisture. The predominant GSL in the tissue of *B. juncea* was sinigrin as reported previously [51,52]. Sinigrin concentration estimated for *B. juncea* (above-ground) was 11 mmol m^−2^. In comparison, Doheny-Adams et al. [51] found a higher concentration of 16–24 mmol m^−2^ under field conditions. Isothiocyanate-release potential is dependent on GSL concentrations, which were sub-optimal in the present study. In contrast, total GSL concentrations as high as 93, 69 and 61 µmol g^−1^ biomass have been detected previously in *B. juncea*, *R. sativus* and *E. sativa*, respectively, under field conditions [33].

Biofumigants grown during summer conditions are exposed to higher UV intensity, longer daylight hours and higher temperatures. These factors are known to increase the production of GSL in brassica tissues [53]. Ngala et al. [33] showed how summer grown brassica crops (*B. juncea*, *E. sativa* and *R. sativus*) produced higher concentrations of GSL in the summer when compared to being overwintered. The high biomass biofumigants, when chopped and incorporated, are then likely to produce ITC at sufficiently effective concentrations. The average maximum temperature for September and October 2018 was recorded as 17.6 °C and 14.5 °C whereas in the previous five-year period (2013–2017), the average maximum temperature in September and October were 18.2 °C and 15.1 °C, respectively. Moreover, the average minimum temperature in September 2018 was 8.8 °C and in October 2018 was 6.1 °C which were 0.6 and 1.6 degrees, respectively, lower than the average for the previous five years [45]. Overall, these two months, when the brassica crops were growing, were slightly cooler compared to the past years. Although the average maximum temperature for August 2018 (21.3 °C) was a degree higher than the average for the 2013–2017 period, the late sowing (13 August 2018) meant that most of the higher temperatures and longer daylight hours were missed. It would have been better if the cover crops were sown in the first week of August which would have probably resulted in higher biomass and higher GSL concentration in the brassica tissues. Moreover, the sun hours in November 2018 (50 h) were lower than the average sun hours for November (61 h) for 2013–2017 period [45]. The lower sun hours in the month, when brassica crops were to be incorporated, might have caused low GSL concentrations.

Estimation of ITC-release potential based on GSL concentration and biomass [46] indicates that sinigrin in *B. juncea* shoots in the present study would produce AITC concentrations at 40 nmol g^−1^ soil, assuming a soil bulk density of 1.4 g cm^−3^ and incorporation to 20 cm. However, this concentration is estimated assuming complete conversion of sinigrin in above-ground tissue to AITC, whereas practically 1% ITC release-efficiency has been reported [46]. Hence, true AITC concentrations were likely to be even lower. In previous in vitro assays, AITC ED_50_ for *F. graminearum* was found to be 99 mg L^−1^ [27], which is equivalent to 998 nmol ml^−1^. This suggests that the potential AITC in *B. juncea* plots (≤40 nmol g^−1^ soil) were very low compared to effective AITC concentrations. Requirement of higher ITC concentrations has also been reported for other ITC such as, methyl ITC concentrations of 517 to 1294 nmol g^−1^ soil are estimated to be required for soil sterilisation [54]. 

Incorporation of *B. oleracea* var. *caulorapa* L. resulted in a mean *F. graminearum* DNA and DON in maize more than 50% lower compared to fallow. Although the effect was not significant, it does suggest a weak biofumigation effect may have occurred. Growth of *B. oleracea* var. *caulorapa* L. was patchy and lower (fresh wt. 20 t ha^−1^) than the other brassicas. A greater biomass of this brassica might have suppressed *F. graminearum* growth more efficiently and subsequently decreased DON concentrations in maize significantly. Fan et al. [55] demonstrated inhibitory effect of *B. oleracea* var. *caulorapa* L. against mycelial growth of *F. graminearum* under in vitro conditions. They reported that 10 g of powdered frozen tissue per Petri dish inhibited the mycelial growth by 70% on day 4 declining to 51% on day 7. In addition to its suppressive effect against fungi [55], *B. oleracea* var. *caulorapa* L. has been found effective in controlling nematodes too. Mashed leaves of *B. oleracea* var. *caulorapa* L. reduced population density of the nematode, *Meloidogyne incognita* (infecting cowpea plant) by 78–80%, whereas reduction by metam sodium was 43–65% in pot experiments [56]. *Brassica oleracea* var. *caulorapa* L. appears to be a potential biofumigant for suppressing *F. graminearum* in field, hence further investigation on the biofumigation effect of this brassica using different cultivars is recommended. *Fusarium graminearum* DNA content in maize was significantly reduced by *R. sativus*. This is in agreement with findings of a pot experiment where macerated tissue of *R. sativus* at a rate of 10 g kg^−1^ soil significantly reduced *F. graminearum* DNA content in soil [57]. In the same study, macerated tissue of *R. sativus* (5 g per Petri dish) also reduced mycelial growth of *F. graminearum* by 17–19%. The reduction in *F. graminearum* DNA content in maize obtained with *R. sativus* may also be attributed to “partial biofumigation”. The thick roots of *R. sativus*, giving a high under-ground biomass, might have released high concentrations of GSL that were probably hydrolysed by myrosinase activity of soil microorganisms [58,59], thus producing biofumigation effect. Ngala et al. [33,60] observed partial biofumigation under a growing *R. sativus* crop, causing suppression of the potato cyst nematode *Globodera pallida* in glasshouse and field conditions.

The present study failed to identify a significant suppression of *F. graminearum* inoculum in soil, and disease and DON contamination in maize, however some of the observed reductions in *F. graminearum* DNA and DON warrant further research. Future studies on optimising agronomy and increasing GSL concentration in brassicas are recommended to achieve potentially successful biofumigation effect. For example, approaches to maximise biomass production, such as, under sowing in the previous crop would allow more time for brassicas to grow and probably result in higher biomass and greater GSL concentration in the brassica tissues. Selection of brassica with the most suitable GSL profile and concentrations, and good biomass are important factors affecting the outcome of biofumigation. Therefore, to achieve a successful biofumigation effect, the approach needs to be optimised considering environmental factors, such as, temperature and moisture content of soil, as well as establishment and growth of the biofumigant crop.

## Figures and Tables

**Table 1:** Brassica cover crops used in a field experiment to assess the effect of biofumigation on *Fusarium graminearum* in a wheat-maize rotation.

Brassica Species	Common Name	Cultivar	Seed Rate	Supplier
*Brassica juncea*	Indian mustard	Brons	8 kg ha^−1^	RAGT Seeds UK
*Brassica carinata*	Ethiopian mustard	Cappucchino	15 kg ha^−1^	RAGT Seeds UK
*Raphanus sativus*	Oilseed radish	Bokito	20 kg ha^−1^	RAGT Seeds UK
*Raphanus sativus*	Oilseed radish	Terranova	20 kg ha^−1^	RAGT Seeds UK
*Eruca sativa*	Rocket	Trio	10 kg ha^−1^	RAGT Seeds UK
*Brassica oleracea* var. *caulorapa* L.	Kohlrabi	Kolibri	0.5 kg ha^−1^	Elsoms, UK

**Table 2:** Shoot and root biomasses of brassica cover crops grown in a field experiment to assess the effect of biofumigation on *Fusarium graminearum* in a wheat-maize rotation.

Brassica	Biomass (t ha^−1^)					
	Fresh Weight			Dry Weight		
	Shoot	Root	Total	Shoot	Root	Total
*Brassica juncea* ‘Brons’	48.4 (5.6) ^a^	4.8 (0.8)	53.3 (6.2)	3.9 (0.7)	0.7 (0.2)	4.6 (0.8)
*Brassica carinata* ‘Cappucchino’	69.5 (16.5)	4.3 (0.7)	73.8 (17)	6.4 (1.6)	0.9 (0.2)	7.3 (1.7)
*Raphanus sativus* ‘Bokito’	79.0 (14.7)	10.4 (2.4)	89.4 (15.3)	6.3 (1.3)	1.1 (0.2)	7.4 (1.3)
*Raphanus sativus* ‘Terranova’	74.0 (22)	10.5 (3.1)	84.6 (23)	5.6 (1.8)	1.0 (0.3)	6.5 (2.0)
*Eruca sativa* ‘Trio’	42.8 (14.4)	2.4 (0.7)	45.3 (15.0)	3.6 (1.1)	0.4 (0.1)	4.0 (1.2)
*Brassica oleracea* var. *caulorapa* L. ‘Kolibri’	19.8 (8.0)	0.9 (0.2)	20.7 (8.2)	2.6 (1.0)	0.2 (0.1)	2.9 (1.1)

^a^ n = 6, numbers in parentheses represent the standard error of the mean.

**Table 3:** Type and concentration of glucosinolates in freeze-dried tissue of brassicas grown in a field experiment to assess the effect of biofumigation on *Fusarium graminearum* in a wheat-maize rotation.

Glucosinolate (µmol g^−1^ freeze-dried tissue)	*B. juncea* ‘Brons’		*B. carinata* ‘Cappucchino’		*R. sativus* ‘Bokito’		*R. sativus* ‘Terranova’		*E. sativa* ‘Trio’		*B. oleracea* var. *caulorapa* L. ‘Kolibri’	
	Shoot	Root	Shoot	Root	Shoot	Root	Shoot	Root	Shoot	Root	Shoot	Root
Glucoberin	2.25 (0.32) ^a^	2.17 (0.38)	2.01 (0.63)	1.89 (0.39)	2.33 (0.74)	2.22 (0.53)	2.35 (0.41)	2.25 (0.75)	2.06 (0.58)	2.18 (0.49)	2.24 (0.21)	1.83 (0.50)
Progoitrin	0.42 (0.13)	0.37 (0.08)	0.34 (0.23)	0.25 (0.17)	0.42 (0.31)	0.54 (0.39)	0.33 (0.22)	0.34 (0.27)	2.08 (1.49)	1.65 (1.02)	0.39 (0.27)	0.18 (0.13)
Sinigrin	26.39 (12.42)	19.97 (12.33)	13.06 (6.21)	4.81 (3.24)	-	-	-	-	-	-	-	-
Gluconapin	0.20 (0.14)	0.14 (0.17)	-	-	-	-	-	-	-	-	-	-
Glucobrassicin	0.17 (0.07)	0.32 (0.04)	0.65 (0.30)	0.43 (0.31)	1.43 (0.79)	1.54 (1.48)	2.05 (1.29)	0.78 (0.67)	0.38 (0.38)	0.05 (0.07)	1.19 (0.76)	0.91 (0.19)
Gluconasturtiin	1.32 (0.70)	18.04 (3.76)	0.42 (0.29)	6.17 (3.50)	1.12 (0.49)	2.47 (1.74)	1.28 (0.61)	2.38 (2.22)	0.90 (0.42)	2.96 (2.18)	0.52 (0.37)	5.42 (1.75)
Neoglucobrassicin	0.07 (0.03)	0.90 (0.17)	0.11 (0.07)	0.61 (0.44)	-	0.42 (0.38)	0.02 (0.03)	0.12 (0.13)	0.03 (0.02)	0.00	0.53 (0.36)	4.35 (1.82)
Glucoraphanin	-	-	-	-	7.84 (4.79)	1.39 (0.95)	5.24 (4.43)	1.47 (1.11)	7.36 (3.96)	3.94 (2.07)	3.45 (2.72)	1.30 (0.67)
Glucoraphenin	-	-	-	-	0.45 (0.11)	-	0.35 (0.19)	-	-	-	-	-
4 hydroxy glucobrassicin	-	0.04 (0.05)	-	-	-	-	-	0.02 (0.03)	-	-	-	0.27 (0.12)
Glucoraphasatin	-	-	-	-	2.43 (1.69)	14.90 (9.77)	3.45 (2.60)	17.60 (10.92)	-	-	-	-
Glucoalyssin	-	-	-	-	-	-	-	-	0.50 (0.21)	0.24 (0.21)	-	-
Glucoerucin	-	-	-	-	-	-	-	-	7.03 (3.65)	12.28 (7.30)	0.14 (0.16)	3.80 (1.71)
4-mercaptobutyl	-	-	-	-	-	-	-	-	4.89 (2.04)	1.26 (1.94)	-	-
unknown	-	-	-	-	-	-	-	-	0.53 (0.37)	-	-	-
unknown	-	-	-	-	-	-	-	-	0.93 (0.70)	-	-	-
Total glucosinolates	30.83 (13.76)	41.91 (15.63)	16.59 (7.62)	14.19 (7.31)	16.02 (8.66)	23.47 (14.65)	15.06 (7.33)	24.96 (15.10)	26.69 (12.33)	24.57 (12.69)	8.46 (4.55)	18.06 (6.22)

^a^ n = 4, numbers in parentheses represent the standard error of the mean.

**Table 4:** Percentage incidence of *Fusarium graminearum* recovered in oat inoculum post-incorporation of brassicas. Sachets containing *Fusarium graminearum*-inoculated blind oat spikes, representing artificial crop debris, were buried in plots post-incorporation of brassica cover crops and removed from the field plots after 8 and 16 weeks.

Treatments	% Incidence *Fusarium graminearum* Recovered in Oat Inoculum	
	8 Weeks Post-Incorporation	16 Weeks Post-Incorporation
Fallow	95	98
Fallow uncultivated	95	97
*Brassica juncea* ‘Brons’	98	97
*Brassica carinata* ‘Cappucchino’	96	95
*Raphanus sativus* ‘Bokito’	96	94
*Raphanus sativus* ‘Terranova’	95	95
*Eruca sativa* ‘Trio’	94	94
*Brassica oleracea* var. *caulorapa* L. ‘Kolibri’	97	97

## Data Availability

All data generated or analysed during this study are included in the published article.
